# A prospective study of age trends of high-risk human papillomavirus infection in rural China

**DOI:** 10.1186/1471-2334-14-96

**Published:** 2014-02-21

**Authors:** Le-Ni Kang, Philip E Castle, Fang-Hui Zhao, Jose Jeronimo, Feng Chen, Pooja Bansil, Jing Li, Wen Chen, Xun Zhang, You-Lin Qiao

**Affiliations:** 1 Cancer Institute and Hospital, Chinese Academy of Medical Sciences and Peking Union Medical College, 17 Panjiayuan Lane, Beijing, 100021, China; 2 Global Cancer Initiative, 100 Radcliff Drive, Chestertown, MD, 21620, USA; 3 PATH, 2201 Westlake Avenue, Suite 200, Seattle, WA, 98121, USA

**Keywords:** Human papillomavirus, Infection, Age, Risk factor, Cohort effect

## Abstract

**Background:**

In China, high-risk human papillomavirus (HR-HPV) prevalence is unexpectedly high in older women, but the possible reasons have not been well studied yet. This study investigated the age trends of HR-HPV infection in a prospective study.

**Methods:**

A total of 7397 women aged 25-65 years without cervical precancer or cancer were evaluated during 2010-2011 with a stratified sample of 2791 women re-evaluated after one year. Test results for *care*HPV and *care*HPV16/18/45 were used to describe the HR-HPV prevalence, incidence and clearance. Risk factors associated with HR-HPV infections were explored using a logistic regression model.

**Results:**

The overall HR-HPV prevalence was 13.1% at baseline, with a peak of 19.3% in women aged 55-59 years. The prevalence of HR-HPV (p for trends < 0.001), HPV16/18/45 (p for trends = 0.002), and HR-HPV other than HPV16/18/45 (p for trends = 0.002) generally increased with increasing age. Number of infections that cleared was generally greater than number of incident infections within age groups. One-year clearance rate decreased with increasing age (p for trends < 0.001), however, incidence rate was unrelated to age *(*p for trends = 0.159). Risk factors that associated with HR-HPV infection differed between younger and older women.

**Conclusions:**

The greater HR-HPV prevalence in older versus younger women in rural China may be explained by a cohort effect, higher than expected incidence, and/or poorer clearance at older age.

## Background

Previous studies of age-specific high-risk human papillomavirus (HR-HPV) prevalence have demonstrated substantial variability across geographical regions [1-3]. Typically, women within a few years of their age of sexual debut were observed to have the highest HR-HPV prevalence. A second peak of HR-HPV prevalence around the age of menopause has been observed in some populations [4,5], while absent in others [6,7].

Due to the lack of well-organized, nation-wide HPV-based cervical cancer screening programmes, the age distribution of HPV prevalence in China has not been well demonstrated, and results from population-based studies also differed from each other [8-12]. More recently, a pooled analysis reported a pronounced increase of HR-HPV prevalence in women around the age of menopause in rural China [13]. However, almost all mentioned studies were cross-sectional in nature (i.e., no longitudinal follow-up), limiting the interpretation of age-specific HR-HPV prevalence in China.

In particular, persistent infection with HR-HPV is the necessary cause of cervical cancer and its precancerous lesions such as cervical intraepithelial neoplasia grade 2 or 3 (CIN2/3) [14-16]. One-year HR-HPV persistence strongly predicts which infection will continue to persist [17] and progress to CIN2/3 [18]. In the U.S., evidence of one-year HR-HPV persistence results in referral to colposcopy [19]. World Health Organization (WHO) is now considering 6-month persistent infection as a surrogate endpoint for HPV vaccine trials.

We therefore wanted to further characterize the age-specific patterns of HR-HPV infections in rural China. Using data from a multi-center study of lower-cost molecular HPV tests in rural China, we described the prevalence and short-term (one-year) dynamics of HR-HPV infection as measured by *care*HPV (Qiagen, Gaithersburg, MD, USA), a DNA test for a pool of 13 carcinogenic HPV genotypes and one possible carcinogenic HPV genotype, which had been evaluated in multiple countries [20,21], and a research-use only DNA test for a pool of HPV16,18, and 45 (HPV16/18/45) made available for the *care*HPV platform (“*care*HPV16/18/45”). We also investigated risk factors for prevalence, incidence, and one-year clearance of HR-HPV.

## Methods

### Study population and procedures

A total of 7541 women aged 25- to 65-year living in rural villages in Shanxi, Henan and Jiangxi provinces were enrolled from October 2010 to August 2011 in a study called “Screening Technologies to Advance Rapid Testing for Cervical Cancer Prevention–Utility and Program Planning (START-UP)” Project. Participant recruitment processes and study procedures were described elsewhere [22]. Briefly, women were considered to be eligible if they: 1) had not been previously diagnosed with cervical cancer; 2) had a cervix; 3) were not pregnant; 4) were physically able to undergo routine cervical cancer screening; and 5) were able to provide informed consent. Each participant provided a written informed consent and a staff-administered questionnaire survey was conducted in a private room. Six screening tests were performed on each participant, including OncoE6™ (Arbor Vita Corporation, Fremont, CA, USA), *care*HPV and Hybrid Capture 2 (HC2) (QIAGEN, Gaithersburg, MD, USA) tested on both self- and clinician-collected specimens and visual inspection with acetic acid (VIA). Woman who were tested positive by any of the six tests or tested negative by all six tests but was selected as a part of ~10% random sample underwent a rigorous colposcopic evaluation using a biopsy protocol as previously described [23]. The primary histological diagnoses were done by two pathologists in CICAMS until reaching an agreement. A U.S. pathologist independently reviewed each initial biopsy or surgical specimen diagnosed as CIN2+, and any discordant diagnoses were settled from discussions with the Chinese pathologists [22].

**Figure 1:**
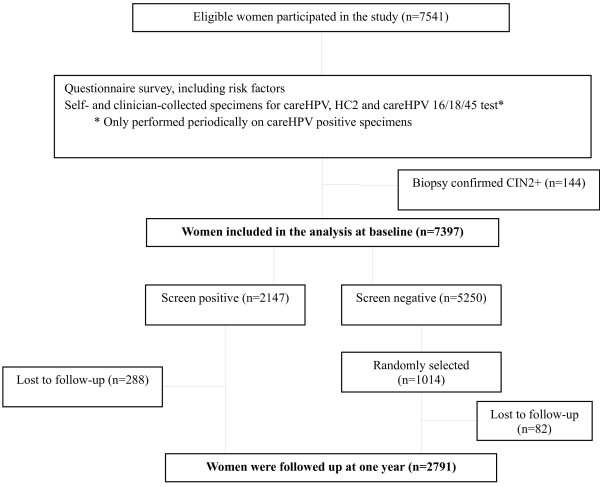
**Flowchart of inclusion and exclusion of the study participants.** At baseline, 144 women were excluded because of biopsy confirmed CIN2+, with 7397 women in the baseline analysis. 288 of the 2147 baseline screen positives were lost to follow-up and 82 of the 1014 randomly selected baseline screen negatives were lost to follow-up, which added up to 2791 women in the follow-up analysis. CIN2+:cervical intraepithelial neoplasia grade 2 or above.

One hundred and forty four women with histology confirmed CIN2+ at baseline were excluded with 7397 women in this analysis. Of the 2147 screen-positive women, 1859 (86.6%) returned for one-year follow-up; of the 5250 screen-negative women, 1014 were randomly selected for one-year follow-up, of whom, 932 (91.9%) returned in a year (Figure 1). All follow-up procedures were the same as the baseline screening without any questionnaire survey.

This study is part of the START-UP Project which was approved by the PATH and Cancer Institute and Hospital, Chinese Academy of Medical Sciences (CICAMS) institutional review boards (IRBs).

### HPV DNA testing

*care*HPV was used to detect HPV DNA in both self- and clinician-collected specimens. *care*HPV is a CE Marked and Chinese Food and Drug (CFDA)-approved signal-amplification DNA assay that uses antibody capture of HPV DNA and RNA probe hybrids with chemiluminescent signal detection. Same as HC2 [24], the relative light unit/cutoff ratio (RLU/CO) is provided as a semi-quantitative measurement of HPV viral load. All *care*HPV-positive specimens were also tested periodically in a batch-wise fashion for HPV16, 18, and 45 by *care*HPV16/18/45.

### Statistical analysis

The prevalence of overall HR-HPV and HPV16/18/45 were demonstrated directly by *care*HPV and *care*HPV16/18/45 results, while the prevalence of HR-HPV other than HPV16/18/45 (other HR-HPV) was inferred as *care*HPV-positive but *care*HPV16/18/45-negative.

The overall HR-HPV prevalence at baseline was standardized by using the world standard population that was reported by the WHO in 2001 [25]. Age-specific prevalence of overall HR-HPV, HPV16/18/45 and other HR-HPV at baseline were simply presented by numbers and rates. Cochran-Armitage trend test was used to test the age trends. Univariate and multivariate logistic regression models were used to calculate the crude and adjusted odds ratios (ORs) of risk factors with 95% confidence intervals (CIs) presented by Wald ϰ^2^ statistics. The HPV status in the successive visits were used to demonstrate incidence (-/+) and clearance (+/-). The number of incidence was adjusted for approximately 20% sampling fraction of baseline screen-negative women. McNemar’s ϰ^2^ test was used to compare the estimated number of incidence and clearance. The adjusted prevalence at follow-up was determined by the estimated number of incidence and clearance.

The results were confirmed by using HC2 outcomes (data not shown). To increase the analytic sensitivity, these analyses were redone by combining the *care*HPV and HC2 results (i.e., HPV positive was defined by either *care*HPV positive or HC2 positive).

SPSS 17.0 (SPSS Inc., Chicago, USA) was used to analyze the data. Statistical significance was assessed by two-tailed tests with an α level of 0.05.

## Results

The median, mean, and age range of the participants were 44, 44.5, and 25-65 years, respectively; 27.5% of the participants were menopausal at baseline, and the mean age at menopause was 48.7 years. Most women were Han ethnicity (99.9%), farmers (75.5%), currently married (97.1%), and of less than 12 years’ education (98.2%). Most women reported that they did not smoke (99.6%) or drink alcohol (91.5%). Most women have self-reported only one sexual partner in their lifetime (87.3%).

Overall, 969 of 7397 women (13.1%, 95% CI: 12.3%-13.9%) were HR-HPV positive at baseline, and the positive rates varied among study sites (12.8% for Yangcheng, 11.9% for Xinmi, and 15.4% for Tonggu). The prevalence of HR-HPV age-standardized to the world’s standard population was 14.2% (95% CI: 13.4%-15.0%). The crude prevalence of HPV16/18/45 was 2.9% (22.3% of all HR-HPV) and of other HR-HPV was 10.2%.

Risk factors associated with the HR-HPV infection are described in Table 1. Univariate analysis showed that age, household income, marital status, age at first menstruation period, age at first sexual intercourse, number of lifetime sex partners, number of sex partners in the past 6 months, age at first pregnancy, number of live births, and menopausal status were significantly associated with HR-HPV infection. In the multivariate model that was adjusted for study sites, education levels and oral contraceptive history, women who were not currently married (OR = 1.50, 95% CI: 1.07-2.12), younger at sex initiation (≤19 vs. ≥23, OR = 1.23, 95% CI: 1.00-1.51), had more than one lifetime sex partners (OR = 1.30, 95% CI: 1.07-1.58), and postmenopause (OR = 1.47, 95% CI: 1.26-1.71) were significantly more likely to have HR-HPV infections. Number of sex partners in the past 6 months was not found to be the independent risk factor for HR-HPV infections (Multivariate analysis: p = 0.086).

**Table 1:** Risk factors associated with overall HPV infection at baseline

**Variables**	**N (%)**	**HPV positive, n (%)**	**Univariate**
			**OR (95% CI)**
**Age**			***p*** **< 0.001**
25-29	326 (4.4)	43 (13.2)	1.0
30-34	631 (8.5)	69 (10.9)	0.81 (0.54-1.21)
35-39	1330 (18.0)	153 (11.5)	0.86 (0.60-1.23)
40-44	1555 (21.0)	188 (12.1)	0.91 (0.64-1.29)
45-49	1425 (19.3)	172 (12.1)	0.90 (0.63-1.29)
50-54	906 (12.2)	126 (13.9)	1.06 (0.73-1.54)
55-59	846 (11.4)	163 (19.3)	**1.57** (1.09-2.26)
60-65	378 (5.1)	55 (14.6)	1.12 (0.73-1.72)
**Household income (Yuan)**^**§**^			***p*** **= 0.044**
≤3000	2609 (35.4)	373 (14.3)	1.0
3001-5000	2254 (30.6)	268 (11.9)	**0.81** (0.68-0.96)
≥5001	2502 (34.0)	323 (12.9)	0.89 (0.76-1.04)
**Marital status**			***p*** < 0.001*
Married	7180 (97.1)	920 (12.8)	1.0
Others	217 (2.9)	49 (22.6)	**1.99** (1.43-2.75)
**Age at first menstruation period (years)**^**§**^			***p*** **= 0.007**
≤14	2654 (35.9)	323 (12.2)	1.0
15-16	2686 (36.4)	336 (12.5)	1.03 (0.88-1.22)
≥17	2047 (27.7)	309 (15.1)	**1.28** (1.09-1.52)
**Age at first sexual intercourse (years)**^**§**^			***p*** < 0.001*
≤19	1499 (20.3)	252 (16.8)	1.0
20-22	4022 (54.4)	487 (12.1)	**0.68** (0.58-0.80)
≥23	1874 (25.3)	230 (12.3)	**0.69** (0.57-0.84)
**No. of lifetime sex partners**^**§**^			***p*** = 0.001*
1	6460 (87.3)	815 (12.6)	1.0
≥2	936 (12.7)	154 (16.5)	**1.36** (1.13-1.65)
**No. of sex partners in the past 6 months**			***p*** **= 0.006**
0	763 (10.3)	121 (15.9)	1.0
1	6553 (88.6)	831 (12.7)	**0.77** (0.63-0.95)
≥2	81 (1.1)	17 (21.0)	1.41 (0.79-2.50)
**Age at first pregnancy (years)**^**§**^			***p*** **= 0.001**
≤20	1954 (26.6)	302 (15.5)	1.0
21-23	3553 (48.4)	427 (12.0)	**0.75** (0.64-0.88)
≥24	1834 (25.0)	227 (12.4)	**0.77** (0.64-0.93)
**No. of live births**^**§**^			***p*** **< 0.001**
0-2	5438 (73.5)	661 (12.2)	1.0
≥3	1958 (26.5)	307 (15.7)	**1.34** (1.16-1.56)
**Menopausal status**^**§**^			***p*** < 0.001*
No	5363 (72.5)	629 (11.7)	1.0
Yes	2033 (27.5)	339 (16.7)	**1.51** (1.31-1.74)

**NOTE:** *Risk factors of statistical significance in multivariate analysis which was adjusted for study sites, education levels and oral contraceptive history.^**§**^Missing values were excluded in the analysis. Bold type indicates statistical significance (*p* < 0.05). HPV = human papillomavirus; OR = odds ratio; CI = confidence interval.

Risk factors associated with HR-HPV infections have also been stratified by tertiles of age (i.e., 25-40, 41-48 and 49-65 years), with the prevalence of 11.4%, 12.4%, and 15.8%, respectively (Table 2). Household income, marital status, age at first menstruation period and age at first pregnancy were found to be associated with HR-HPV infection in women aged 25-40 years. By comparison, age at first intercourse showed significant association in 41 to 48-year-old women; and lifetime number of sex partners and menopausal status were significantly associated with having HR-HPV in women aged 49-65 years.

**Table 2:** Risk factors associated with overall HR-HPV infection at baseline by age group

**Age-group**	Risk factors*	**HPV positive, n (%)**	**Multivariate**
			**OR (95% CI)**
**25-40 (n = 2587)**	**Household income (Yuan)**^**§**^		***p*** **= 0.023**
	≤3000	91 (11.2)	1.0
	3001-5000	79 (10.1)	0.96 (0.69-1.34)
	≥5001	125 (12.7)	**1.41** (1.04-1.92)
	**Marital status**		***p*** **= 0.007**
	Married	289 (11.3)	1.0
	Others	7 (29.2)	**3.48** (1.40-8.66)
	**Age at first menstruation period (years)**^**§**^		***p*** **= 0.027**
	≤14	140 (10.5)	1.0
	15-16	110 (11.6)	1.06 (0.80-1.40)
	≥17	46 (15.3)	**1.65** (1.14-2.40)
	**Age at first pregnancy (years)**^**§**^		***p*** **= 0.004**
	≤20	97 (14.5)	1.0
	21-23	136 (11.0)	0.77 (0.57-1.03)
	≥24	55 (8.5)	**0.54** (0.38-0.78)
**41-48 (n = 2530)**	**Age at first sexual intercourse (years)**^**§**^		***p*** **< 0.001**
	≤19	74 (21.1)	1.0
	20-22	173 (10.9)	**0.49** (0.35-0.67)
	≥23	66 (11.2)	**0.52** (0.36-0.77)
**49-65 (n = 2280)**	**No. of lifetime sex partners**^**§**^		***p*** **= 0.006**
	1	306 (15.0)	1.0
	≥2	54 (22.0)	**1.58** (1.14-2.20)
	**Menopausal status**^**§**^		***p*** **= 0.008**
	No	48 (11.3)	1.0
	Yes	311 (16.8)	**1.55** (1.12-2.15)

NOTE: *Only those risk factors of statistical significance in multivariate analysis which was adjusted for study sites, education level, oral contraceptive history, number of sex partners in the past 6 months, number of live births were shown. Age was categorized by tertiles at baseline. Bold type indicates statistical significance (*p* < 0.05).^**§**^Missing values were excluded in the analysis. HPV = human papillomavirus; OR = odds ratio; CI = confidence interval.

**Figure 2:**
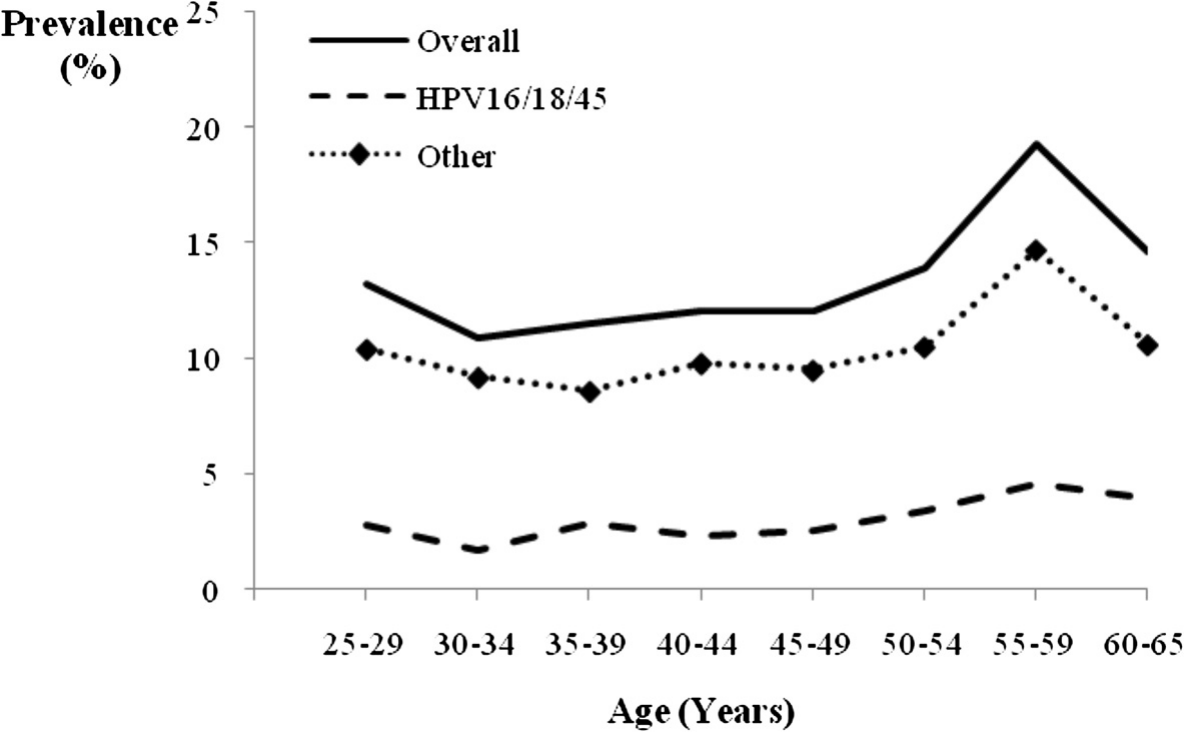
**Age group-specific prevalence of any high-risk HPV, any HPV16, 18, and/or 45 (HPV16/18/45), and high-risk HPV other than HPV16/18/45 at baseline.** Figure 2 Symbols: (bold line) Overall, (dash line) HPV16/18/45, (dot-dash line with solid diamond) Other.

Figure 2 illustrates the age-specific prevalence of overall HR-HPV, HPV16/18/45 and other HR-HPV at baseline by 5-year intervals. The age-specific prevalence of overall HR-HPV between 25 and 54 years formed a “U-shaped curve”, with a prevalence of 13.2% in women aged 25-29 years and 13.9% in women aged 50-54 years and a nadir of 10.9% in women aged 30-34 years. The prevalence peaked in the 55-59 year age group at 19.2% and then decreased to 14.6% in the 60-65 year age group. A similar age pattern was observed for other HR-HPV. In contrast, the prevalence of HPV16/18/45 appeared to rise steadily with age p for trend = 0.002) but there was no spike in the prevalence in the 55-59 year age group. The HR-HPV prevalence performed on the definition of either *care*HPV positive or HC2 positive showed similar age trends.

The average follow-up time of the stratified sample of women was 11.7 (SD = 0.3) months. The crude and sampling fraction-adjusted HR-HPV prevalence at follow-up was 20.6% and 11.1%, respectively. The net change in HR-HPV infection is demonstrated in Table 3. The total and age-specific numbers of new HPV infection were less than those of HPV clearance, except for women aged 35-39 and 60-65 years, although most of the differences were not statistically significant.

**Table 3:** Incidence, clearance and prevalence of overall HR-HPV infections at one-year follow-up by age

**Age group**	**Crude prevalence, n/N (%)**	**Adjusted prevalence, n/N (%)**	**Adjusted incidence, n/N (%)**	**Clearance, n/N (%)**	**P**^**a**^
25-29	15/102 (14.7)	28/302 (9.3)	21/269 (7.8)	26/33 (78.8)	0.560
30-34	35/242 (14.5)	51/598 (8.5)	24/541 (4.4)	30/57 (52.6)	0.497
35-39	91/494 (18.4)	141/1 275 (11.1)	82/1 145 (7.2)	71/130 (54.6)	0.419
40-44	100/593 (16.9)	163/1 503 (10.8)	105/1 339 (7.8)	106/164 (64.6)	1.000
45-49	97/552 (17.6)	125/1 390 (9.0)	57/1 235 (4.6)	87/155 (56.1)	**0.015**
50-54	91/345 (26.4)	108/877 (12.3)	43/765 (5.6)	47/112 (42.0)	0.752
55-59	100/315 (31.7)	116/801 (14.5)	33/667 (4.9)	51/134 (38.1)	0.063
60-65	46/148 (31.1)	56/363 (15.4)	22/316 (7.0)	13/47 (27.7)	0.175
**Total**	575/2 791 (20.6)	788/7 109 (11.1)	387/6 277 (6.2)	431/832 (51.8)	0.133

**NOTE:** Since only a portion of baseline screen negative women were followed up at one-year, incidence was adjusted for the sampling fraction. It was calculated as follows: ((1/sampling fraction) * (Number of baseline screen negatives who tested careHPV-positive at follow-up) + (Number of baseline screen positives with careHPV-negative who tested careHPV-positive at follow-up)) / ((Number of baseline screen negatives) + (Number of baseline screen positives with careHPV-negative who were followed up)). The clearance was simply calculated by: (Number of cleared) / (Number of baseline careHPV-positive who were followed up). The adjusted prevalence at follow up was determined as: ((Number of baseline careHPV-positives who were not cleared) + (Number of incidence)) / Total population. Age groups were defined on the basis of age at baseline. a: the McNemar x^2^ test is used to test for statistical differences in clearance and incidence; bold type indicates statistical significance (*p* < 0.05).

**Figure 3:**
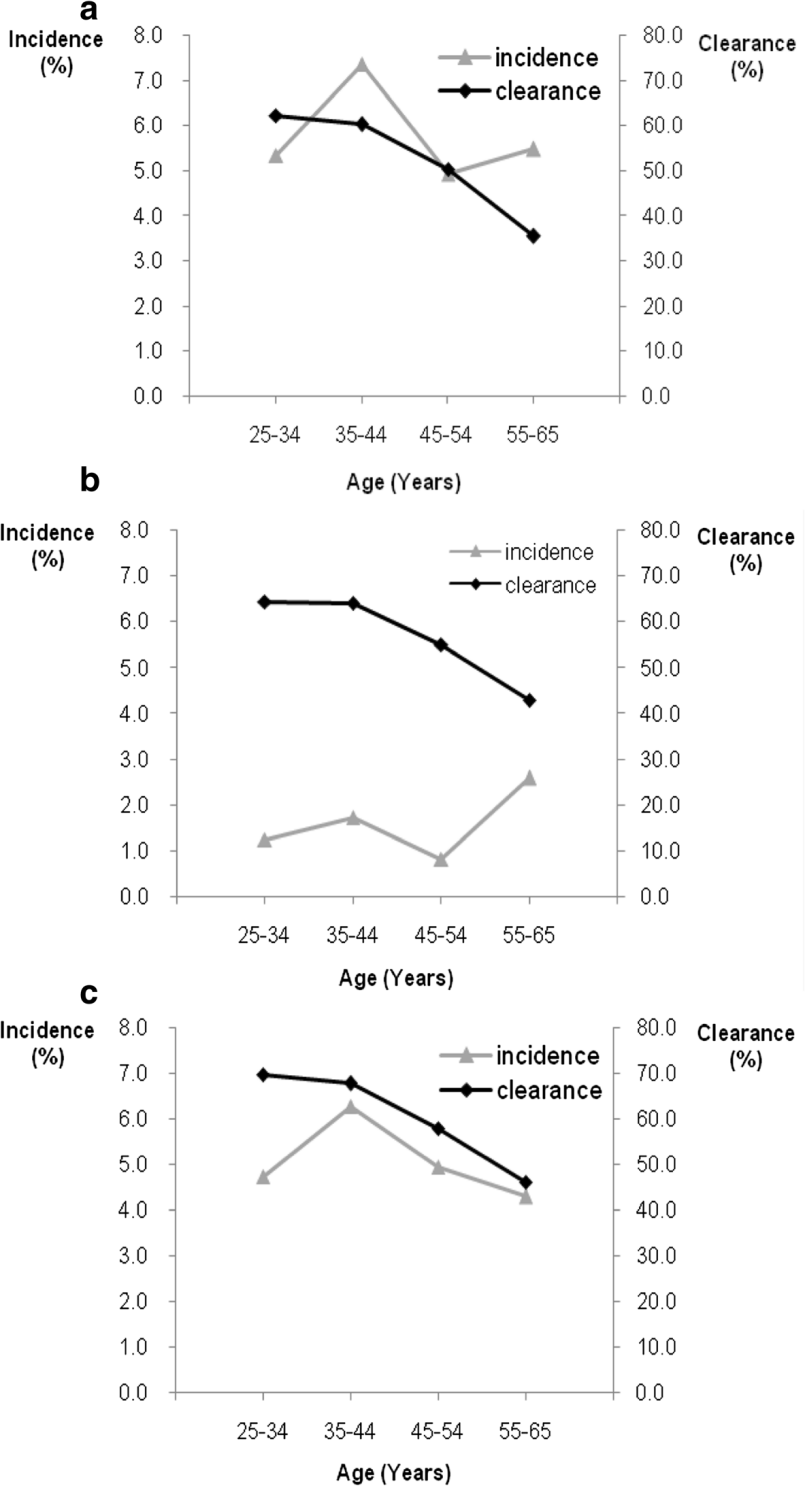
**Incidence and clearance of HPV by age group.** Incidence is adjusted for sampling fraction of baseline screen negatives. Age groups are defined on the basis of age at baseline. **a.** Incidence and clearance of overall HPV infection. **b.** Incidence and clearance of HPV16/18/45 infection. **c.** Incidence and clearance of other HPV infection. Figure 3 Symbols: (gray line with solid triangle) incidence, (black line with solid diamond) clearance.

The age trends of incidence and clearance of overall HR-HPV, HPV16/18/45 and other HR-HPV are illustrated in Figure 3. The clearance of overall HPV, HPV16/18/45 and other HPV declined with increasing age (p for trends < 0.05, for all), while no significant trends for incidence of overall HR-HPV, HPV16/18/45 and other HR-HPV were observed (p for trends > 0.05, for all). The HR-HPV incidence and clearance rates performed on the definition of either *care*HPV positive or HC2 positive showed similar results.

The baseline risk factors of HR-HPV incidence and clearance are also investigated (Table 4). Women who had more than one sex partners in the last 6 months were more likely to have an incident HR-HPV infection at the one-year follow-up (2 vs. 0, OR = 7.80, 95% CI: 2.46-24.75), while older women and women with higher baseline HR-HPV signal strength, a semi-quantitative measure of HR-HPV viral load [26,27], were less likely to clear the infection. Infection of HPV16/18/45 was marginally associated (p = 0.052, OR = 0.70, 95% CI: 0.48-1.00) with less clearance compared to other HR-HPV genotypes.

**Table 4:** Risk factors associated with overall HR-HPV incidence and clearance

**Incidence/Clearance**	Risk factors*		**Univariate**	**Multivariate**
			**OR (95% CI)**	**OR (95% CI)**^**ξ**^
**Incidence (n = 1959)**	**No. of sex partners in the past 6 months**		***p*** **= 0.002**	***p*** **= 0.001**
		0	1.0	1.0
		1	1.01 (0.58-1.76)	1.11 (0.62-1.97)
		2	**6.40** (2.04-20.05)	**7.80** (2.46-24.75)
**Clearance (n = 832)**	**Age (years)**		***p*** **< 0.001**	***p*** **< 0.001**
		25-29	1.0	1.0
		30-34	**0.30** (0.11-0.80)	**0.28** (0.10-0.78)
		35-39	**0.32** (0.13-0.80)	**0.36** (0.14-0.91)
		40-44	0.49 (0.20-1.20)	0.48 (0.19-1.21)
		45-49	**0.34** (0.14-0.84)	**0.35** (0.14-0.89)
		50-54	**0.20** (0.08-0.49)	**0.19** (0.07-0.48)
		55-59	**0.17** (0.07-0.41)	**0.15** (0.06-0.38)
		60-65	**0.10** (0.04-0.30)	**0.11** (0.04-0.33)
	**Education level (years)**		***p*** **= 0.001**	*p* = 0.204
		≤6	1.0	
		7-12	**1.61** (1.22-2.13)	
		≥13	**3.29** (1.03-10.51)	
	**Age at first sexual intercourse (years)**		***p*** **= 0.037**	*p* = 0.496
		≤19	1.0	
		20-22	**1.46** (1.05-2.04)	
		≥23	1.06 (0.72-1.56)	
	**No. of sex partners in the past 6 months**		***p*** **= 0.014**	*p = 0.066*
		0	1.0	
		1	**1.68** (1.08-2.58)	
		2	0.60 (0.20-1.84)	
	**Menopausal status**^**§**^		***p*** **< 0.001**	*p* = 0.433
		No	1.0	
		Yes	**0.41** (0.31-0.55)	
	**No. of live births**^**§**^		***p*** **< 0.001**	*p* = 0.695
		0-2	1.0	
		≥3	**0.57** (0.42-0.76)	
	**Signal strength (RLU/CO)**		***p*** **< 0.001**	***p < 0.001***
		1.00-9.99	1.0	1.0
		10.00-99.99	**0.27** (0.20-0.38)	**0.28** (0.20-0.40)
		≥100.00	**0.43** (0.29-0.62)	**0.40** (0.27-0.59)
	**HPV16/18/45**		***p*** **< 0.001**	*p* = 0.052
		Negative	1.0	1.0
		Positive	**0.56** (0.40-0.78)	0.70 (0.48-1.00)

**NOTE:** *Risk factors were defined on the basis of risk factors at baseline. ^**ξ**^Multivariate logistic model was adjusted for study sites, household income, marital status and oral contraceptive for incidence and clearance. Furthermore, for incidence, VIA and HC2 results at baseline were also adjusted in the multivariate analysis. HPV16/18/45 was put into the model for marginally association. In the multivariate analysis, OR was given only if statistical significant. ^**§**^Missing values were excluded in the analysis. Bold type indicated statistical significant (*p* < 0.05). HPV = human papillomavirus; OR = odds ratio; CI = confidence interval.

## Discussion

This study investigated the age-specific prevalence of overall HR-HPV, HPV16/18/45 and other HR-HPV in 25 to 65-year-old women without cervical precancer and cancer in a prospective study in rural China. It focused on the interpretation for the peak observed in older women. Moreover, we explored the risk factors of HPV incidence and clearance using one-year follow-up data.

The age-standardized prevalence by world population of HR-HPV tested by *care*HPV at baseline in our study was 14.2%, which was comparable to other studies in mainland China [8-10], and other parts of Asia/Australia (~15%) [28]. However, it was lower than a pooled analysis from China (16.8%) [13]. We reported a prevalence of 2.9% for HPV16 and/or 18 and/or 45 types, which was lower than other studies in China [9,10]. These differences are likely caused by different HPV DNA tests and different study populations, such as different geographic areas, different age groups, and different distributions of cervical lesions. We found that the prevalence as measured by *care*HPV was similar to the prevalence as measured by HC2 in our study (p = 0.196), which suggested that the difference between the prevalence in this study and the recent pooled analysis [13] was primarily due to the differences among study populations.

Previous studies in China had observed a second peak of HPV prevalence in older women, although the peak age varied between studies [8-10,13]. In our study, HR-HPV prevalence peaked at 55-59 years, but lacked the “first peak” in younger women. It has been well acknowledged that cumulative risks of 40-50% of HPV acquisition happened within 2 to 3 years of sexual debut [29-32]. Since the average age at first sexual intercourse of our study participants was 21.2 years, the theoretical peak of HPV prevalence should appear in women aged 23-24 years. In addition, Chinese women were less likely to report their premarital and extramarital sexual histories. In that case, we hypothesize that the actually age of sexual initiation may be even younger. However, we only enrolled women aged over 25 years, therefore we have no chance to see the “first peak” as observed in other studies [13]. The one-year prospective data demonstrated a larger number of infections that were cleared versus acquired, which indicated that HR-HPV prevalence should decrease with progressing of age, rather than a peak occurred in older women.

The probable explanations for this HR-HPV peak in older Chinese women could be a cohort effect that leads to increased lifetime exposures, increased HPV incidence ,and/or increased viral persistence in older women [33] versus younger women. We considered each as described below.

### Cohort effects

Before the one-child policy which had been strictly implemented in year 1979 [34], most women got married at an early age and gave a large number of births. Risk factor analysis in our study also found an earlier age at first sexual intercourse and a greater number of live births in 49 to 65-year-old women (data not shown), which may have led to a higher cumulative exposure to HR-HPV. And in some high-quality cancer registries in China, a declining trend in cervical cancer incidence from 1970’s to 1990’s has been observed [35]. This may partly reflect a higher HR-HPV burden in older generations (vs. younger generations according to a report from Sharma et al. [36], which found a positive association between age-standardized HPV prevalence and its square-root age-standardized cervical incidence).

However, one-year follow-up was not sufficient to provide a strong evidence to explain the cohort effect. Long-term follow-up studies with birth cohort analysis are needed to further clarify this effect. With recent changes in sexual norms, we anticipate an upturn in the annual incidence of cervical cancer in China unless widespread secondary prevention through screening, diagnosis, and treatment of precancerous lesions is implemented.

### HPV incidence

We expected to see a decreased trend in the incidence of HR-HPV infection in older women as reported in other studies [33,37], but only observed an independent relation. This unexpected higher incidence in older women may be caused by: 1) new HPV infections acquired by changes in sexual behaviors by either the women or their partners; 2) reactivation of latent HPV infection due to immune senescence [38,39]. No evidence supported the changes in sexual behaviors in older women although that does not rule out that their male partners were having new partners and transmitting HPV to them. However, we noted differences in risk factors associated with HPV infection between women aged 25-40 years and women aged 49-65 years. Number of lifetime sex partners, which may be proxy for cumulative exposure impact [40], was only found to be associated with HPV infection in older women. Greater early exposure to HR-HPV infection and weakened immune response after menopause [41] that led to the re-emergence of latent HPV infections could cause a second HPV peak in older women. Gravitt *et al*. [39] found that there was a second peak around 50-54 years in women with five or more sex partners but not in those with less than four sex partners. Their findings may support that the second peak could be seen in a more generalized population with a relatively higher cumulative probability of HPV infection.

### HPV persistence

Another explanation, a greater HPV persistence in older women, was strongly supported by our data (see Additional file 1: Figure S1). We found an obvious trend of decreasing clearance or increasing persistence of HPV with increasing age. The mechanisms could be: 1) decreased ability to clear recent infections with age-related immune senescence; 2) predominance of long-duration prevalent infections in older women from earlier exposures [33].

Thus, we found that the cause of the second peak in HPV prevalence may be multi-factorial. Despite of the low number of sex partners, the rural Chinese women were usually married at an early age and gave a large number of births. The lifestyles and economic status of the older rural Chinese women were also much different from other populations and younger generations. Their poor nutrition and sanitary condition may cause a poor immune function, which may in turn result in a reduced ability to clear HPV infections and to control/prevent the re-emergence of latent HPV infections.

Our findings agreed with the previous study conducted in Guanacaste, Costa Rica, which also found that HPV infections tended to clear more often than acquire, and persistence increased with age [33]. However, unlike the study in Guanacaste, Costa Rica, we did not observe newly detected infections declining with age.

### Risk factors

We also explored the risk factors for HPV prevalence, incidence and clearance using both univariate and multivariate models. Most of our results were similar to the others [42,43], however, we failed to find the association between the number of sex partners in the past 6 months and HPV prevalence in the multivariate model. This may be explained by the fact that most of the participants reported to have no or one sex partners in the past 6 months. To our knowledge, this may not be entirely accurate as some women may be reluctant to report their actual behaviors.

### Impact for cervical cancer screening

The incidence of HPV16,18, and/or 45 was higher and the clearance was lower in 55 to 65-year-old women, they are of the greatest risk of persisting HPV16/18/45 infections, which cause 75% of cervical cancer worldwide [44]. Women aged 55-65 years may be at the highest risk of progression to cervical precancer or cancer. However, the first large-scaled cervical cancer screening program in rural China launched by Chinese government from 2009 to 2011 using VIA or Pap smear does not cover women older than 60 years [45]. If further studies show these infections representing significant cancer risk in older women, expanding screening to older ages should be considered in the future nationwide program in China.

### Limitations

This analysis has some limitations. First of all, convenience sampling was used in recruiting, the participants may not be perfectly representative. Second, we only followed up ~20% of the screen negatives, even with adjustment, the estimated incidence and prevalence at follow-up might differ somewhat from the actual values. Whereas, we conducted a random sampling to get the list of screen-negative women, so the bias was minimized. Third, we did not test for the specific HPV type, so that we could not get the precise status of HPV incidence or clearance. Given that multiple infections typically represent 20-30% of all HR-HPV infections and are often more common in younger women [46], we likely underestimated both one-year clearance and incidence. However, we granted the same patterns of clearance and incidence for HPV16/18/45 infections as the overall HR-HPV infections, which may reflect a relatively low percentage of women with co-infections of the three types. We only use a pooled DNA test for 14 certain and probable HR-HPV types to generally describe the age-group specific patterns of HR-HPV, which is relevant to cervical cancer screening and the risk factors associated.

We used clinical tests for HPV, which may have missed some lower viral load HPV infections that are not strongly associated with CIN2+. To address this issue, we conducted an additional analysis by defining HPV positive as either *care*HPV or HC2 tested positive to increase the analytic sensitivity. And we observed similar results.

Another notable limitation was the short follow-up time. As a consequence, some infections persisted for a year but would have later cleared, while some infections that appeared to clear were testing errors and would have tested positive subsequently. However, based on the results from a meta-analysis which found the median duration of HR-HPV detection was 10.9 months in those HPV-positive women with normal cytology [47], we believe that measuring the pattern over a year was a good surrogate for the longer-term persistence. Previous studies have shown that one-year HPV persistence can strongly predict longer-term persistence [17] and CIN2+ [47-49].

## Conclusions

Our study observed an increase of HR-HPV prevalence in older women in rural China. The probable explanations could be: 1) cohort effect; 2) higher than expected incidence; and/or 3) poorer clearance/greater persistence of HR-HPV at older ages. Long-term prospective studies with frequent follow-up intervals by HPV genotyping are needed to verify the conclusions from this study.

## Competing interests

PEC has received commercial HPV tests for research at a reduced or no cost from Roche, QIAGEN, Norchip, and mtm. He is a paid consultant for BD, GE Healthcare, and Cepheid, and has received a speaker’s honorarium from Roche. He is a paid consultant for Immunexpress on sepsis diagnostics. He is compensated as a member of a Merck Data and Safety Monitoring Board for HPV vaccines. JJ was the director of the study and received all the tests used in the study as a donation from the manufacturing companies (QIAGEN and Arbor Vita Corporation). All other authors have no competing interests.

## Authors’ contributions

PEC, JJ and YLQ contributed to conception and design of the study. All authors were involved in the study implementation. LNK, FHZ, JJ, FC, JL, WC, XZ assisted in the data collection; LNK, JJ, PB, JL contributed to the data management. PEC, LNK designed the analysis; all authors were involved in data analysis and interpretation. LNK, PEC, and YLQ drafted manuscript. All authors read and approved the final manuscript.

## Pre-publication history

The pre-publication history for this paper can be accessed here:

http://www.biomedcentral.com/1471-2334/14/96/prepub

## Supplementary Material

Additional file 1: Figure S1. Age group-specific, one-year persistence of any high-risk HPV, any HPV16, 18, and/or 45 (HPV16/18/45), and high-risk HPV other than HPV16/18/45. Additional file 1: Figure S1 Symbols: (bold line) Overall, (dash line) HPV16/18/45, (the dot-dash line with solid diamond) Other. Click here for file

## References

[B1] Schiffman M Castle PE Jeronimo J Rodriguez AC Wacholder S Human papillomavirus and cervical cancer Lancet 2007 14 890 907 10.1016/S0140-6736(07)61416-0 17826171

[B2] Clifford GM Gallus S Herrero R Muñoz N Snijders PJ Vaccarella S Anh PT Ferreccio C Hieu NT Matos E Worldwide distribution of human papillomavirus types in cytologically normal women in the International Agency for Research on Cancer HPV prevalence surveys: a pooled analysis Lancet 2005 14 991 998 10.1016/S0140-6736(05)67069-9 16168781

[B3] Franceschi S Herrero R Clifford GM Snijders PJ Arslan A Anh PT Bosch FX Ferreccio C Hieu NT Lazcano-Ponce E Variations in the age-specific curves of human papillomavirus prevalence in women worldwide Int J Cancer 2006 14 2677 2684 10.1002/ijc.22241 16991121

[B4] Ferreccio C Prado RB Luzoro AV Ampuero SL Snijders PJ Meijer CJ Vaccarella SV Jara AT Puschel KI Robles SC Population-based prevalence and age distribution of human papillomavirus among women in Santiago, Chile Cancer Epidemiol Biomarkers Prev 2004 14 2271 2276 15598792

[B5] Herrero R Castle PE Schiffman M Bratti MC Hildesheim A Morales J Alfaro M Sherman ME Wacholder S Chen S Epidemiologic profile of type-specific human papillomavirus infection and cervical neoplasia in Guanacaste, Costa Rica J Infect Dis 2005 14 1796 1807 10.1086/428850 15871111

[B6] Peto J Gilham C Deacon J Taylor C Evans C Binns W Haywood M Elanko N Coleman D Yule R Cervical HPV infection and neoplasia in a large population-based prospective study: the Manchester cohort Br J Cancer 2004 14 942 953 15292939 10.1038/sj.bjc.6602049 PMC2409880

[B7] Kjaer SK Breugelmans G Munk C Junge J Watson M Iftner T Population-based prevalence, type- and age-specific distribution of HPV in women before introduction of an HPV-vaccination program in Denmark Int J Cancer 2008 14 1864 1870 10.1002/ijc.23712 18661520

[B8] Dai M Bao YP Li N Clifford GM Vaccarella S Snijders PJ Huang RD Sun LX Meijer CJ Qiao YL Human papillomavirus infection in Shanxi Province, People’s Republic of China: a population-based study Br J Cancer 2006 14 96 101 10.1038/sj.bjc.6603208 16773069 PMC2360486

[B9] Li LK Dai M Clifford GM Yao WQ Arslan A Li N Shi JF Snijders PJ Meijer CJ Qiao YL Human papillomavirus infection in Shenyang City, People’s Republic of China: a population-based study Br J Cancer 2006 14 1593 1597 10.1038/sj.bjc.6603450 17088908 PMC2360733

[B10] Wu RF Dai M Qiao YL Clifford GM Liu ZH Arslan A Li N Shi JF Snijders PJ Meijer CJ Human papillomavirus infection in women in Shenzhen City, People’s Republic of China, a population typical of recent Chinese urbanisation Int J Cancer 2007 14 1306 1311 10.1002/ijc.22726 17417776

[B11] Liu SS Chan KY Leung RC Chan KK Tam KF Luk MH Lo SS Fong DY Cheung AN Lin ZQ Prevalence and risk factors of Human Papillomavirus (HPV) infection in southern Chinese women - a population-based study PLoS One 2011 14 e19244 10.1371/journal.pone.0019244 21559276 PMC3086888

[B12] Yu XW Zhang XW Wang L Li F Xu J Status of human papillomavirus infection in the rural female population in Northwestern China: an observational study J Low Genit Tract Dis 2013 14 17 22 10.1097/LGT.0b013e31825707ab 22885647

[B13] Zhao FH Lewkowitz AK Hu SY Chen F Li LY Zhang QM Wu RF Li CQ Wei LH Xu AD Prevalence of human papillomavirus and cervical intraepithelial neoplasia in China: a pooled analysis of 17 population-based studies Int J Cancer 2012 14 2929 2938 10.1002/ijc.27571 22488743 PMC3435460

[B14] Walboomers JM Jacobs MV Manos MM Bosch FX Kummer JA Shah KV Snijders PJ Peto J Meijer CJ Muñoz N Human papillomavirus is a necessary cause of invasive cervical cancer worldwide J Pathol 1999 14 12 19 10.1002/(SICI)1096-9896(199909)189:1<12::AID-PATH431>3.0.CO;2-F 10451482

[B15] Boulet GA Horvath CA Berghmans S Bogers J Human papillomavirus in cervical cancer screening: important role as biomarker Cancer Epidemiol Biomarkers Prev 2008 14 810 817 10.1158/1055-9965.EPI-07-2865 18398022

[B16] Bosch FX Lorincz A Muñoz N Meijer CJ Shah KV The causal relation between human papillomavirus and cervical cancer J Clin Pathol 2002 14 244 265 10.1136/jcp.55.4.244 11919208 PMC1769629

[B17] Plummer M Schiffman M Castle PE Maucort-Boulch D Wheeler CM ALTS Group A 2-year prospective study of human papillomavirus persistence among women with a cytological diagnosis of atypical squamous cells of undetermined significance or low-grade squamous intraepithelial lesion J Infect Dis 2007 14 1582 1589 10.1086/516784 17471427

[B18] Koshiol J Lindsay L Pimenta JM Poole C Jenkins D Smith JS Persistent human papillomavirus infection and cervical neoplasia: a systematic review and meta-analysis Am J Epidemiol 2008 14 123 137 10.1093/aje/kwn036 18483125 PMC2878094

[B19] Saslow D Solomon D Lawson HW Killackey M Kulasingam SL Cain J Garcia FA Moriarty AT Waxman AG Wilbur DC American Cancer Society, American Society for Colposcopy and Cervical Pathology, and American Society for Clinical Pathology screening guidelines for the prevention and early detection of cervical cancer Am J Clin Pathol 2012 14 516 542 10.1309/AJCPTGD94EVRSJCG 22431528

[B20] Gage JC Ajenifuja KO Wentzensen N Adepiti AC Stoler M Eder PS Bell L Shrestha N Eklund C Reilly M Effectiveness of a simple rapid human papillomavirus DNA test in rural Nigeria Int J Cancer 2012 14 2903 2909 10.1002/ijc.27563 22473652 PMC3404249

[B21] Qiao YL Sellors JW Eder PS Bao YP Lim JM Zhao FH Weigl B Zhang WH Peck RB Li L A new HPV-DNA test for cervical-cancer screening in developing regions: a cross-sectional study of clinical accuracy in rural China Lancet Oncol 2008 14 929 936 10.1016/S1470-2045(08)70210-9 18805733

[B22] Zhao FH Jeronimo J Qiao YL Schweizer J Chen W Valdez M Lu P Zhang X Kang LN Bansil P An evaluation of novel, lower-cost molecular screening tests for human papillomavirus in rural China Cancer Prev Res (Phila) 2013 14 938 948 10.1158/1940-6207.CAPR-13-0091 23878179

[B23] Pretorius RG Zhang WH Belinson JL Huang MN Wu LY Zhang X Qiao YL Colposcopy directed biopsy, random cervical biopsy, and endocervical curettage in the diagnosis of cervical intraepithelial neoplasia II or worse Am J Obstet Gynecol 2004 14 430 434 10.1016/j.ajog.2004.02.065 15343217

[B24] Gravitt PE Burk RD Lorincz A Herrero R Hildesheim A Sherman ME Bratti MC Rodriguez AC Helzlsouer KJ Schiffman M A comparison between real-time polymerase chain reaction and hybrid capture 2 for human papillomavirus DNA quantitation Cancer Epidemiol biomarkers Prev 2003 14 477 484 12814990

[B25] Ahmad OB Boschi-Pinto C Lopez AD Murray CJ Lozano R Inoue M Age standardization of rates: a new WHO standard. Series: No . 31 2001 Geneva: World Health Organization Press 10 [www.who.int/healthinfo/paper31.pdf]

[B26] Moodley JR Constant D Hoffman M Salimo A Allan B Rybicki E Hitzeroth I Williamson AL Human papillomavirus prevalence, viral load and pre-cancerous lesions of the cervix in women initiating highly active antiretroviral therapy in South Africa: a cross-sectional study BMC Cancer 2009 14 275 10.1186/1471-2407-9-275 19664216 PMC2739859

[B27] Safaeian M Herrero R Hildesheim A Quint W Freer E Van Doorn LJ Porras C Silva S González P Bratti MC Comparison of the SPF10-LiPA system to the hybrid capture 2 assay for detection of carcinogenic human papillomavirus genotypes among 5,683 young women in Guanacaste, Costa Rica J Clin Microbiol 2007 14 1447 1454 10.1128/JCM.02580-06 17344361 PMC1865890

[B28] Smith JS Melendy A Rana RK Pimenta JM Age-specific prevalence of infection with human papillomavirus in females: a global review J Adolesc Health 2008 14 4 Suppl S5 S25 18809145 10.1016/j.jadohealth.2008.07.009

[B29] Collins S Mazloomzadeh S Winter H Blomfield P Bailey A Young LS Woodman CB High incidence of cervical human papillomavirus infection in women during their first sexual relationship BJOG 2002 14 96 98 10.1111/j.1471-0528.2002.01053.x 11845815

[B30] Winer RL Lee SK Hughes JP Adam DE Kiviat NB Koutsky LA Genital human papillomavirus infection: incidence and risk factors in a cohort of female university students Am J Epidemiol 2003 14 218 226 10.1093/aje/kwf180 12543621

[B31] Rodriguez AC Burk R Herrero R Hildesheim A Bratti C Sherman ME Solomon D Guillen D Alfaro M Viscidi R The natural history of human papillomavirus infection and cervical intraepithelial neoplasia among young women in the Guanacaste cohort shortly after initiation of sexual life Sex Transm Dis 2007 14 494 502 17237737 10.1097/01.olq.0000251241.03088.a0

[B32] Moscicki AB Management of adolescents who have abnormal cytology and histology Obstet Gynecol Clin North Am 2008 14 633 643 10.1016/j.ogc.2008.09.004 19061822 PMC2766533

[B33] Castle PE Schiffman M Herrero R Hildesheim A Rodriguez AC Bratti MC Sherman ME Wacholder S Tarone R Burk RD A prospective study of age trends in cervical human papillomavirus acquisition and persistence in Guanacaste, Costa Rica J Infect Dis 2005 14 1808 1816 10.1086/428779 15871112

[B34] Wang C History of the Chinese family planning progrom: 1970-2010 Contraception 2012 14 563 569 10.1016/j.contraception.2011.10.013 22176797

[B35] Wei K Wang Y Liang Z An analysis of incidence with cervical cancer in 1970 ~ 2007 in Zhongshan city, Guangdong province China Cancer 2012 14 495 497 in Chinese

[B36] Sharma M Bruni L Diaz M Castellsagué X de Sanjosé S Bosch FX Kim JJ Using HPV prevalence to predict cervical cancer incidence Int J Cancer 2013 14 1895 1900 10.1002/ijc.27835 22965284

[B37] Goodman MT Shvetsov YB McDuffie K Wilkens LR Zhu X Thompson PJ Ning L Killeen J Kamemoto L Hernandez BY Prevalence, acquisition, and clearance of cervical human papillomavirus infection among women with normal cytology: Hawaii human papillomavirus cohort study Cancer Res 2008 14 8813 8824 10.1158/0008-5472.CAN-08-1380 18974124 PMC2727731

[B38] Gravitt PE Rositch AF Silver MI Marks MA Chang K Burke AE Viscidi RP A cohort effect of the sexual revolution may be masking an increase in human papillomavirus detection at menopause in the United States J Infect Dis 2012 14 272 280 23242540 10.1093/infdis/jis660 PMC3532829

[B39] Bosch FX Burchell AN Schiffman M Giuliano AR de Sanjose S Bruni L Tortolero-Luna G Kjaer SK Muñoz N Epidemiology and natural history of human papillomavirus infections and type-specific implications in cervical neoplasia Vaccine 2008 14 Suppl 1 K1 K16 18847553 10.1016/j.vaccine.2008.05.064

[B40] Strickler HD Kirk GD Figueroa JP Ward E Braithwaite AR Escoffery C Drummond J Goebel B Waters D McClimens R HPV 16 antibody prevalence in Jamaica and the United States reflects differences in cervical cancer rates Int J Cancer 1999 14 339 344 10.1002/(SICI)1097-0215(19990129)80:3<339::AID-IJC1>3.0.CO;2-F 9935171

[B41] González P Hildesheim A Rodríguez AC Schiffman M Porras C Wacholder S Piñeres AG Pinto LA Burk RD Herrero R Behavioral/lifestyle and immunologic factors associated with HPV infection among women older than 45 years Cancer Epidemiol Biomarkers Prev 2010 14 3044 3054 10.1158/1055-9965.EPI-10-0645 20952561 PMC3703390

[B42] Lai CH Chao A Chang CJ Chao FY Huang HJ Hsueh S Lin CT Cheng HH Huang CC Yang JE Host and viral factors in relation to clearance of human papillomavirus infection: a cohort study in Taiwan Int J Cancer 2008 14 1685 1692 10.1002/ijc.23679 18623128

[B43] Sellors JW Karwalajtys TL Kaczorowski J Mahony JB Lytwyn A Chong S Sparrow J Lorincz A Incidence, clearance and predictors of human papillomavirus infection in women CMAJ 2003 14 421 425 12591782 PMC143547

[B44] de Sanjose S Quint WG Alemany L Geraets DT Klaustermeier JE Lloveras B Tous S Felix A Bravo LE Shin HR Human papillomavirus genotype attribution in invasive cervical cancer: a retrospective cross-sectional worldwide study Lancet Oncol 2010 14 1048 1056 10.1016/S1470-2045(10)70230-8 20952254

[B45] The Lancet: Women’s health in rural China Lancet 2009 14 358 10.1016/S0140-6736(09)61394-5 19647592

[B46] Méndez F Muñoz N Posso H Molano M Moreno V van den Brule AJ Ronderos M Meijer C Muñoz A Cervical coinfection with Human Papillomavirus (HPV) types and possible implications for the prevention of cervical cancer by HPV vaccines J Infect Dis 2005 14 1158 1165 10.1086/444391 16136457

[B47] Rositch AF Koshiol J Hudgens MG Razzaghi H Backes DM Pimenta JM Franco EL Poole C Smith JS Patterns of persistent genital human papillomavirus infection among women worldwide: a literature review and meta-analysis Int J Cancer 2013 14 1271 1285 10.1002/ijc.27828 22961444 PMC3707974

[B48] Rodríguez AC Schiffman M Herrero R Wacholder S Hildesheim A Castle PE Solomon D Burk R Rapid clearance of human papillomavirus and implications for clinical focus on persistent infections J Natl Cancer Inst 2008 14 513 517 10.1093/jnci/djn044 18364507 PMC3705579

[B49] Castle PE Rodriguez AC Burk RD Herrero R Wacholder S Alfaro M Morales J Guillen D Sherman ME Solomon D Short term persistence of human papillomavirus and risk of cervical precancer and cancer: population based cohort study BMJ 2009 14 b2569 10.1136/bmj.b2569 19638649 PMC2718087

