# Supplementary figures and images for: A prospective study of age trends of high-risk human papillomavirus infection in rural China
Source: BMC Infect Dis. 2014 Feb 21;14:96. doi: 10.1186/1471-2334-14-96 (PMC3936871; doi:10.1186/1471-2334-14-96)

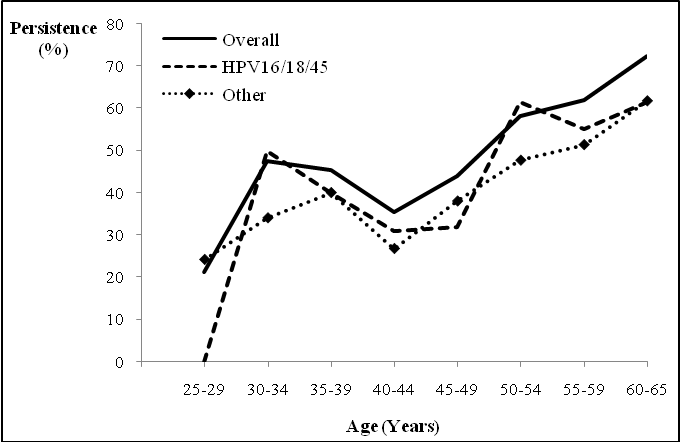

Supplement: Additional file 1: Figure S1. — Age group-specific, one-year persistence of any high-risk HPV, any HPV16, 18, and/or 45 (HPV16/18/45), and high-risk HPV other than HPV16/18/45. Additional file 1: Figure S1 Symbols: (bold line) Overall, (dash line) HPV16/18/45, (the dot-dash line with solid diamond) Other. [file 1471-2334-14-96-S1.doc]
